# Drp1-Dependent Mitochondrial Fission Plays Critical Roles in Physiological and Pathological Progresses in Mammals

**DOI:** 10.3390/ijms18010144

**Published:** 2017-01-13

**Authors:** Chenxia Hu, Yong Huang, Lanjuan Li

**Affiliations:** Collaborative Innovation Center for Diagnosis and Treatment of Infectious Diseases, State Key Laboratory for Diagnosis and Treatment of Infectious Diseases, School of Medicine, First Affiliated Hospital, Zhejiang University, Hangzhou 310058, China; 11318093@zju.edu.cn (C.H.); 21518657@zju.edu.cn (Y.H.)

**Keywords:** dynamin-related protein 1, mitochondria, fission, fusion, mammal

## Abstract

Current research has demonstrated that mitochondrial morphology, distribution, and function are maintained by the balanced regulation of mitochondrial fission and fusion, and perturbation of the homeostasis between these processes has been related to cell or organ dysfunction and abnormal mitochondrial redistribution. Abnormal mitochondrial fusion induces the fragmentation of mitochondria from a tubular morphology into pieces; in contrast, perturbed mitochondrial fission results in the fusion of adjacent mitochondria. A member of the dynamin family of large GTPases, dynamin-related protein 1 (Drp1), effectively influences cell survival and apoptosis by mediating the mitochondrial fission process in mammals. Drp1-dependent mitochondrial fission is an intricate process regulating both cellular and organ dynamics, including development, apoptosis, acute organ injury, and various diseases. Only after clarification of the regulative mechanisms of this critical protein in vivo and in vitro will it set a milestone for preventing mitochondrial fission related pathological processes and refractory diseases.

## 1. Introduction

The mitochondrion is the main organelle for producing adenosine triphosphate (ATP) and acts as a regulator in synthesis of metabolites, phospholipids, heme, and intracellular calcium homeostasis [1]. Imbalanced mitochondrial fission and fusion always lead to mitochondrial structural changes and dysfunction, thus it is critical to develop new methods for conserving the balance between mitochondrial fission and fusion in mammals. Abnormal mitochondrial fusion induces the fragmentation of mitochondria from a tubular morphology into pieces; in contrast, perturbed mitochondrial fission results in the fusion of adjacent mitochondria [2,3]. As a main regulator in mitochondrial fission process, Dynamin-related protein 1 (Drp1) in mammals consists of four different domains: the N-terminal GTP-binding, middle, insert B, and C-terminal GTPase effector (GED) domains [4]; insert B (also known as the variable domain) plays a critical role in the regulative process of mitochondrial fission since it binds the target membrane effectively [4]. This functional protein is termed Dnm1p in yeast but Drp3A/B in plants [5,6]. Two morphologically distinct multimers of Drp1 coexist under physiological conditions in vitro and in vivo; dimers reorganize Drp1, thus resulting in remodeling of the mitochondrial membrane, whereas multimers promote Drp1 GTPase activity and induce mitochondrial fission [7]. Intriguingly, loss of Drp1 triggers genome instability and initiates DNA damage response by disrupting the mitochondrial division and distribution [8]; disturbance consequently results in the reduction of mitochondrial membrane potential and the mitochondrial electron transport chain for energy production [9]. This review mainly focuses on the detailed mechanisms and machinery of Drp1-dependent mitochondrial fission, and we can conclude that Drp1-dependent mitochondrial fission is an intricate process for regulating cellular and organ dynamics, including development, apoptosis, acute organ injury, and various diseases in mammals. In this way, it will provide guidance for regulation of mitochondrial fission in various pathological processes and diseases.

## 2. The Detailed Regulatory Mechanisms of Dynamin-Related Protein 1 (Drp1)-Dependent Mitochondrial Fission

Drp1-dependent mitochondrial fission can be divided into four steps: translocation of Drp1 to the mitochondrial outer membrane (MOM), subsequent higher-order assembly, GTP hydrolysis, and ultimately disassembly [10]. Drp1 binds to receptors in the MOM and forms a functional complex, and a larger oligomer can subsequently be assembled and transported from the cytoplasm to the fission sites [11]. In addition, the endoplasmic reticulum (ER) helps transfer Ca^2+^ into the mitochondria, resulting in recruitment of Drp1 to the mitochondrial surface [12]. However, reactive oxygen species (ROS) can also be upstream initiators of mitochondrial fission, in which case Drp1 can be activated through Cdk1, PKCδ, and calcineurin-mediated pathways [13]. When Drp1 is activated accompanied with reduced diameters in mitochondria, the mitochondrion divides unevenly and generates two daughter organelles with unequal membrane potential [14], while the lengths of Drp1 helical rings along the mitochondria are not significantly altered [15].

In general, when ER starts to encircle the mitochondria, the mitochondria immediately divide and mitochondrial fission factor (Mff)-dependent Drp1 assembly is subsequently initiated [16]. Although there are various isoforms of Drp1 generated by selective splicing of pre-mRNA transcripts, Mff effectively and differentially regulates the activities of Drp1 in a cooperative GTPase-dependent pathway [11]. Mff knockdown has been shown to induce mitochondrial elongation in mammalian cells, whereas overexpression of Mff recruits Drp1 for mitochondrial fragmentation [17]. Premature self-assembly of Drp1 impairs its interactions with Mff, and the abundance of Drp1–Mff heterodimers is significantly increased after removal of the insert B domain of Drp1 [18]. There are two vital receptors in Drp1-dependent mitochondrial fission: mitochondrial elongation factor 1 (MiD51) binds to an adenosine diphosphate (ADP) co-factor, whereas mitochondrial elongation factor 2 (MiD49) physically recruits Drp1 to the mitochondrial surface [19]. However, MiD51 has been demonstrated to promote the recruitment of Drp1 without regulatory elements, such as Mff, mitochondrial fission 1 (Fis1), and mitofusion 2 (Mfn2) [20]. In addition, MiD49 activity has been demonstrated to be more indispensable than Mff and Fis1 [21]. There is still controversy surrounding the function of human Fis1, which uniformly localizes throughout the MOM and improves the activity of Drp1 by competitively binding to MiD51 [22]. Mai et al. have shown that Fis1 knockdown increases the interconnectivity of mitochondrial tubules, whereas overexpression increases the mitochondrial fragmentation [23]. In contrast, Osellame et al. have demonstrated that Drp1 activity is not influenced by silencing or overexpression of human Fis1 [24].

In addition to the regular process of mitochondrial fission, there are a variety of alternative pathways or elements for regulation of Drp1-dependent fission. GTP hydrolysis and Drp1 activity are restrained in mitochondrial division after Drp1 recognizes the head group of phosphatidic acid and two saturated acyl chains of another phospholipid found in the MOM [25]. Drp1 and cardiolipin cooperatively promote membrane constriction and prime fission through co-localization and self-assembly at the membrane bilayer [26]. Actin filaments are able to bind purified Drp1 and interact with Mff, thereby increasing the GTPase activity, whereas inhibiting actin polymerization significantly reduces Drp1-dependent mitochondrial fission [27]. Inhibition of the receptor-interacting protein kinase 1 decreases the level of c-Jun NH_2_-terminal kinase activation and effectively abrogates Drp1 translocation [28]. Although ganglioside-induced differentiation-associated protein 1 induces mitochondrial fragmentation, loss of Drp1 effectively rescues the impairment [29]. Through a similar pathway, loss of Drp1 also protects against Tyrphostin A9-induced mitochondrial filament fragmentation [30].

For better clarification of the regulative mechanisms in mitochondrial fission, we need to have a superficial cognition in the opposite direction of Drp1-dependent mitochondrial fission. During the mitochondrial fusion process, the membranes of two adjacent mitochondria fuse through the formation of hetero- and homo-oligomeric protein complexes [31]. Although the MOM is fused independently of the mitochondrial inner membrane (MIM), they together co-regulate the mitochondrial redistribution progress. Mitochondrial fusion proteins including Mfn1 and Mfn2 are located on the MOM, whereas optic atrophy 1 (Opa1) is located on the MIM, and all three proteins cooperatively regulate mitochondrial morphology and function [32]. Even though overexpression of Mfn1 and Mfn2 is not able to restore the mitochondrial fragmentation and reduce cell viability [33], ubiquitylation of Mfn1 plays critical roles in enhancing cell survival rates under stress conditions [34]. Disassembly of Opa1 oligomers induces neither the remodeling of mitochondrial cristae nor the release of cytochrome c [35,36]. However, Opa1 stabilizes mitochondrial structure and increases mitochondrial respiratory efficiency, thereby eliminating mitochondrial dysfunction and cytochrome c release in ischemic environments [37].

## 3. Post-Translational Modifications of Drp1 in Mitochondrial Fission

Post-translational modifications of Drp1 predominantly include phosphorylation, *S*-nitrosylation, SUMOylation, ubiquitination, and *O*-GlcNAcylation [10]. Because Drp1 phosphorylation is initiated at several sites, it takes part in different mechanisms of various pathological processes.

For better understanding post-translational phosphorylation of Drp1 at different sites, the correlated mechanisms in different mammalian cells or tissues for mitochondrial fission are demonstrated in Table 1. In general, phosphorylation at Ser-637 inhibits Drp1 activity while phosphorylation at Ser-616 activates Drp1 activity; some specific upstream kinases also influence Drp1 function [38]. Moreover, phosphorylation of Drp1 at the same residue is likely to have opposite effects on the mitochondrial fission progress; this may depend on external parameters including cell type, age or status, or on internal parameters. For example, Ca^2+^/calmodulin dependent protein kinase Iα promotes phosphorylation of Drp1 at Ser-637 and increases Drp1 translocation to mitochondria [39]. In contrast, cAMP-dependent protein kinase phosphorylate Drp1 at the same residue effectively inhibits Drp1 activity in a cardiac ischemic injury model [40]. A kinase anchoring protein 1 (AKAP1) promotes phosphorylation of Ser-637, thereby inducing mitochondrial network extension, whereas dephosphorylation of Ser-637 results in programmed necrosis [41,42]. AKAP1 is a scaffold protein that recruits protein kinase A (PKA) and integrates several second messenger cascades to modulate mitochondrial function and associated physiological and pathophysiological outcomes [43]. In addition, Ser-616 can also be phosphorylated by several kinases including the cyclin-dependent kinase (CDK) family [44], ERK1/2 [45], and PKCδ [13]. Adenosine monophosphate-activated protein kinase (AMPK) activation alters the state of Drp1 phosphorylation, and suppresses ER stress and inflammatory cytokine release [46]. PKA suppresses Drp1-dependent mitochondrial fission at different sites including phosphorylation at site Ser-637 or dephosphorylation at site Ser-616 [38,47], while cyclin-B-dependent kinase promoting the phosphorylation of Drp1 at Ser-616 does not directly affect GTPase activity, but facilitates the increasing of mitochondrial fragmentation [48]. The PKA/AKAP1 signaling pathway and protein phosphatase 2A (PP2A)/Bβ2 signaling pathway exert opposite effects on the phosphorylation of Drp1 at the Ser-656 site: PKA/AKAP1 promotes the phosphorylation of Drp1-mediated dendrite occupancy and enhances dendritic outgrowth but paradoxically decreases the synapse number and density, while PP2A/Bβ2 promotes dephosphorylation of Drp1-mediated depolarized mitochondria to cease dendritic outgrowth but augment synapse formation [49]. There are also other phosphorylation sites for regulation of Drp1 activity. Oxidative stress leads to mitochondrial fragmentation by promoting phosphorylation of Ser-579 in human Drp1 isoform 3 [50]; glycogen synthase kinase (GSK)3β-mediated phosphorylation at Ser-693 dramatically decreases GTPase activity but does not affect Drp1 inter- or intra-molecular interactions [51].

After establishing the basic functions of different phosphorylated sites as mentioned above, multiple studies focus on how the phosphorylation of Drp1 at different sites influences related pathological processes in vivo and in vitro. In diabetes animal models or patients, hyperglycemia stimulates the recruitment of Drp1 in podocytes and endothelial cells by directly facilitating the phosphorylation of Drp1 at Ser-600 in mice or Ser-637 in humans [52]. In smooth muscle cell, cdk1/cyclin B mediates the phosphorylation of Ser-616 and leads to pulmonary arterial remodeling [54]; glucagon-like peptide-1 stimulates mitochondrial fusion but decreases vascular smooth muscle cell dedifferentiation through enhanced phosphorylation of Drp1 at Ser-637 site [39]. Dominant-negative forkhead box O3a enhanced phosphorylation of Drp1 at the Ser-637 site and upregulated maladaptive cardiac atrophy genes in cardiac myocytes via modulation of calcium homeostasis-mediated mitochondrial apoptosis and autophagy [58]. In anoxia–reoxygenation injury-induced cardiomyocytes, ROS production, mitochondrial fission, and phosphorylation of Drp1 Ser-616 are increased but are accompanied by the downregulation of phosphorylated Ser-637 [13]. Although angiotensin II or hydrogen peroxide induce smooth muscle cells to proliferate and migrate, decreasing the phosphorylation of Drp1 at Ser-616 neutralizes these effects [55]. Furthermore, Ca^2+^-dependent protein kinase Iα promotes the phosphorylation of Ser-637 in human Drp1 isoform 1, but also promotes the phosphorylation of Ser-600 in human Drp1 isoform 3, thus consequently inducing mitochondrial fragmentation in cultured hippocampal neurons [39]. Wnt-5a upregulates intracellular and mitochondrial calcium, thus treated hippocampal cells demonstrate increased phosphorylation of Drp1 Ser-616 but decreased phosphorylation of Drp1 at Ser-637 [64]. As a chemical inhibitor of cyclin-dependent kinases, roscovitine increased oligomerization and mitochondrial translocation of Drp1; in contrast, Cyclin-dependent kinase 5 modulates mitochondrial morphology via inhibitory phosphorylation of Drp1 Ser-616 in post-mitotic neurons in vitro [60]. All these regulators help to modulate Drp1 through phosphorylation at different sites and change the status of cells in vitro or tissue function in vivo. As an important step in the regulation of Drp1, SUMOylation stimulate stable binding of Drp1 to the MOM, and its activity is significantly affected [67]. SUMOylation of Drp1 activates the ER-mediated calcium flux, the remodeling of mitochondrial cristae, and the release of cytochrome c [68]. When Drp1 ubiquitylation and proteasomal degradation are inhibited during interphase, synthesis of cytokines is diminished, and unequal mitochondrial distributions occur rapidly [69]. The overexpression of mitochondrial SUMO E3 ligase substantially promotes mitochondrial fission [70], whereas ectopic expression of Sentrin/SUMO-specific protease (SENP)-5 plays opposite roles by promoting the deSUMOylation of Drp1 [71,72]. Furthermore, loss of SENP3 prolongs Drp1 SUMOylation and suppresses cell apoptosis [73].

When the activity of *N*-acetyl-glucosaminidase is ablated, *O*-GlcNAcylation of Drp1 at Thr-585 and Thr-586, within the variable domain, is significantly enhanced and consequently increases mitochondrial fragmentation in cardiomyocytes [74]. In addition, *S*-nitrosylation is activated within the GED domain at Cys644, and then upregulates the production of Drp1 oligomers and GTPase activity in neurons, thereby leading to the pathology of Huntington’s disease [64]. However, this conclusion is controversial, because Bossy et al. have demonstrated that *S*-nitrosylation of Drp1 exerts no influence on Alzheimer’s disease and does not induce oligomerization of Drp1 [75].

## 4. Drp1-Dependent Mitochondrial Fission and Development In Vivo and In Vitro

Increasing numbers of studies show that Drp1-dependent mitochondrial fission influences the growth and health of cells, from primordial germ cells to functional terminal cells, in vivo and in vitro (Table 2). Aged mice demonstrate reduced Drp1 activity and defective organelle morphogenesis in oocytes, mainly through impaired Ca^2+^ signaling and intercellular communication [76]. In the muscle tissue of these mice, although there is no difference in the expression levels of mitochondrial fission and fusion proteins, the Mfn2-to-Drp1 ratio is significantly increased, and the intermyofibrillar mitochondria are longer and more branched [77]. It was also showed that loss of Drp1 induced the death of mice by embryonic day 12.5 [78]; cardiac-specific Drp1 knockout (KO) mice had impaired left ventricular function and died within 13 weeks through suppression of autophagic flux [79]. As a Drp1 peptide inhibitor, P110 is neuroprotective by inhibiting the p53-mediated apoptotic pathways in neurons of 1-methyl-4-phenyl-1,2,3,6-tetrahydropyridine (MPTP) animal models [80]. Drp1-null mice failed to undergo developmentally regulated apoptosis during neural tube formation in vivo and died at embryonic day 11.5, although without altered intracellular ATP levels in these models [81]. On the other hand, overexpression of Drp1 in a transgenic mouse line results in inhibition of the growth hormone pathway and postnatal muscle growth, as well as reduced mitochondrial DNA (mtDNA) quantity [82].

Mitochondria, the dynamic energy powerhouses of the cell, play vital roles in a multitude of cellular processes, including differentiation and cell survival [88]. In addition, growth factor erv1-like (Gfer) helps maintain pluripotency in murine ESCs, and preserves survival ability via the modulation of Drp1 [88]. To further clarify the mechanisms of the Gfer-mediated mitochondrial fission process, Todd et al. studied knockdown Gfer in ESCs and found that the expression levels of pluripotency marker, embryoid body formation, and cell survival all decreased [89]. Although knockdown of Drp1 does not affect mitochondrial function, proliferation ,and pluripotency of ESCs [83], Drp1 knockdown negatively influences terminal differentiation of ESCs, particularly in neurogenetic differentiation by downregulation of pluripotency-associated genes, although it is not critical for mitochondria biogenesis for ESC proliferation [83]. In addition, the loss of Drp1 also augments the cyclin E pool, thus attenuating cell proliferative rates in mouse embryonic fibroblasts (MEFs) seeded at low density. However, these cells exhibit aberrant proliferation when they are seeded at high density [84]. The dominant-negative form also helps to rescue the impaired myogenic differentiation potency in myogenic precursor cells [85]. Through inhibition of cyclin-dependent kinase-mediated Drp1 activation, mdivi-1 decreases aerobic metabolism, calcium flux, and proliferation of ductal smooth muscle cells [86]. Drp1 is demonstrated to be required for cell survival since inhibition of Drp1 significantly increased mitochondrial length and caused cell death in cortical neurons [87].

## 5. Drp1-Dependent Mitochondrial Fission and Apoptosis

**Figure 1:**
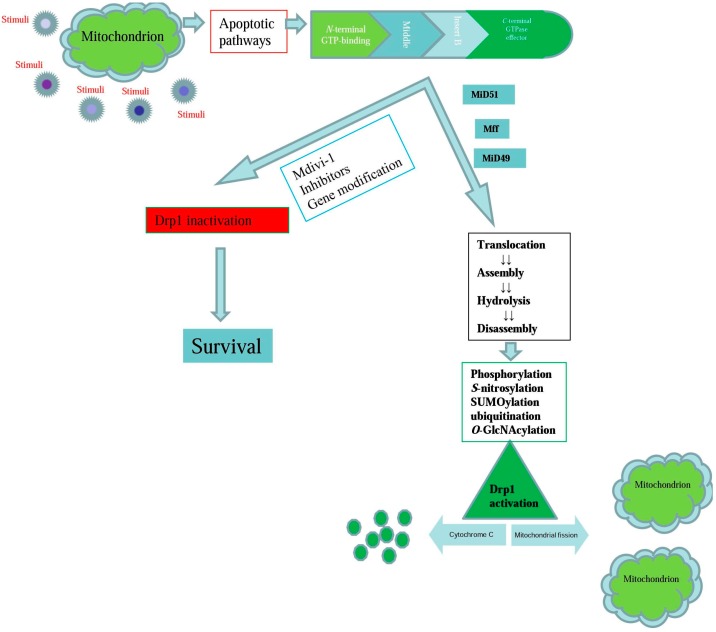
Various stress conditions induce the initiation of apoptotic pathways, mitochondrial fragmentation and concomitant apoptosis. Then Drp1-dependent mitochondrial fission is mediated by translocation of Drp1 to the mitochondrial outer membrane (MOM), subsequent higher-order assembly, GTP hydrolysis, and ultimately disassembly. The effective formation of Drp1 is regulated through post-translational modifications, which results in cytochrome c release and mitochondrial fragmentation. However, interferences inhibit the mitochondrial fission pathway and improve survival rates, even under stressful conditions. Mff, mitochondrial fission factor; MiD51, mitochondrial elongation factor 1; MiD49, mitochondrial elongation factor 2.

After Drp1-dependent mitochondrial fission is altered directly or indirectly, apoptosis can be altered through different signaling pathways (Figure 1). Various stress conditions induce the translocation of Drp1, thus rapidly resulting in excessive mitochondrial fragmentation and concomitant apoptosis [90]. The apoptosis-related signals are transferred to the ER, and a complex with Bap31 is formed at the interface of the ER and mitochondria [91]. Human Fis1 is also involved in the apoptosis signaling pathway by transporting apoptotic signals to the ER [91]. During apoptosis, the cooperation of Bax and fission-related proteins regulates the mitochondrial fission progress [92]. Mitochondrial fission often occurs adjacent to nucleoids, and the clustering of nucleoids often leads to the formation of enlarged or elongated mitochondria; consequently, cytochrome c release is suppressed under apoptotic stimuli [93]. Despite the above relationship between apoptosis and Drp1-dependent mitochondrial fission, Bik stimulation leads to mobilization of intra-mitochondrial cytochrome c and cooperatively activates Bax independently of Drp1 activity [94]. 

Since there are various routes to regulate Drp1 activity directly or indirectly, these treatments highly influence the apoptotic outcomes. Pseudo-phosphorylation of Drp1 improves the resistance to various pro-apoptotic elements; in contrast, dephosphorylation of Drp1 significantly increases cell vulnerability [95]. Inhibition of Drp1 also prevents the release of soluble Opa1, thereby affecting mitochondrial cristae remodeling and cytochrome c release [36]. Although mdivi-1 is demonstrated to exert strong cytoprotective effect on various cell types in vivo and in vitro, it also takes part in resisting proliferative and cytotoxic effects in tumors and immortalized cells via reducing cell mitosis and enhancing cell apoptosis [96]; the cytotoxic effect of mdivi-1 is also correlated with the Bax/Bak pathway in MEFs [96]. Mdivi-1 extensively elongates the mitochondria and increases the apoptosis rate, although Drp1 phosphorylation at Ser-637 is gradually decreased in differentiated myoblasts [97]. Because of the essential role of Bax in cytochrome c release, loss of Bax absolutely inhibits Drp1-dependent mitochondrial fission and decreases the apoptosis rate [98]. However, whereas mitofusins and the loss of Drp1 inhibit Bax insertion and activation, they are not able to affect the translocation of Bax to mitochondria [99]. Depletion of death-associated protein 3, which is specifically located in the matrix of mitochondria, dramatically decreases the phosphorylation of Drp1 at Ser-637 and leads to inhibited autophagy and increased apoptotic sensitivity in cells [65]. ABT-737, a non-peptidic Bcl2/X(L) inhibitor, decreases oxygen consumption rates (OCRs) and improves the release of cytochrome c; although Drp1 KO MEFs are less sensitive than wild-type MEFs, the expression of Bax and the related apoptosis rates are unaltered [100]. Astaxanthin markedly promotes myofibroblast apoptosis by upregulating the levels of Drp1 and apoptosis-associated genes, including Bcl2 and p53 [101]. The suppressor of cytokine signaling 6 mediates apoptosis by forming a complex with Drp1 and phosphoglycerate mutase 5 (PGAM5) and attenuating phosphorylation of Drp1 [102]. Both Debcl and Drp1 are downstream of Buffy in the Jun Kinase pathway and trigger an enhanced ROS production and mitochondrial fragmentation during Rbf1-induced apoptosis [103]. Betanodavirus B2 increases the production of hydrogen peroxide (H_2_O_2_) and leads to cell death in vitro and in vivo via Drp1 activation [104]. Although Bcl2/adenovirus E1B interacting protein 1 increase Drp1 translocation, this effect is fully abrogated by Bcl2 overexpression [105]. Apoptosis signaling pathways are correlated with the regulation of mitochondrial morphology, and the inhibition or promotion of Drp1 activity effectively influences the effects of apoptotic stimuli in vivo and in vitro.

## 6. Drp1-Dependent Mitochondrial Fission and Pathological Processes

Because Drp1 participates in various important physiological processes in mammals, the altered expression of this protein significantly affects the results of the related processes. Especially in a variety of ischemia or ischemia-reperfusion (IR) models in vivo and in vitro, for example, inhibition of Drp1 selectively blocks mitophagy without affecting mitochondrial biogenesis, but Drp1-dependent mitophagy is triggered to remove damaged mitochondria during the early phase of ischemic-induced injury [106]. Under inhibition of Drp1 with Drp1-K38A, transduced cardiomyocytes show a significant decrease of OCRs with an inconspicuous alteration of ATP production after IR [107]. In IR-induced cardiomyocytes, mdivi-1 preserves the mitochondrial structure and significantly reduces the myocardial infarction area [54,108]. Tacrolimus (FK506) treatment prior to ischemia-reperfusion (IR) prevented the specific dephosphorylation and preserved cardiac function via reduction of ROS, improvement of left ventricular developed pressure and lowered left ventricular end diastolic pressure in adult rat hearts [59]. Pim-1 activity inhibits Drp1 compartmentalization and protects against the disturbance of mitochondrial morphology by increasing phosphorylation at Ser-637 in response to simulated ischemia cardiomyocytes [109]. Drp1 inhibition by mdivi-1 not only resists IR-induced injury in the heart but also protects other somatic cells from IR injury by blocking the mitochondrial apoptosis pathway [110,111]. Furthermore, necrostatin-1 inhibits cell death in the ischemia-injured rat tubular cell line [112]. Although IR induced injury significantly activates phosphorylated Drp1 at Ser-616, mild hypothermia suppresses the alteration and preserves neural cells integrity for reducing rates of neuronal necrosis and apoptosis [61]. In addition, an agonist of peroxisome proliferator-activated receptor-γ decreases phosphorylation of Drp1 and demonstrates resistance against neuronal IR-induced injury [113]. Through attenuating mitochondrial translocation of Drp1, PTEN-induced novel kinase 1 (PINK1) significantly ameliorates cell death and inhibits the ischemia-induced mitochondrial fission in neurons [62]. Furthermore, mdivi-1 significantly attenuated neurotoxicity and pre-existing deficits in PTEN-induced putative kinase-1 deletion and 1-methyl-4-phenyl-1,2,3,6-tetrahydropyridine mouse models [114].

After incubation of retinal ganglion (RGC)-5 cells under high hydrostatic pressure for several days in vitro, mitochondrial cristae depletion, ATP reduction and Drp1 translocation are triggered for mitochondrial fission, accompanied by irreversible functional impairments [115]. These alterations have also been observed in glaucomatous mice, whereas the suppression of this progress rescues impaired retinas by maintaining mitochondrial integrity [116]. Cyclosporine A demonstrates nephrotoxicity in renal tubular cells by increasing the levels of fission proteins but decreasing the levels of fusion proteins [117]. Rhabdomyolysis induces kidney dysfunction by upregulating Drp1 activity and ROS production but down-regulating ATP levels, whereas mdivi-1 maintains the function by eliminating release of Bax and cytochrome c [118]. Furthermore, mdivi-1 immediately prevents radiation-induced mitochondrial fragmentation and attenuates induced high apoptosis rate [119]. As an effective inhibitor of Drp1 activity, mdivi-1 has considerable effects in animal models. Although acetaminophen-induced cell death can be suppressed by reduced expression of inhibiting receptor interacting protein kinase 3 and mdivi-1, the protective effects were lost after one day in vivo [120]. Nrf2 KO mice were susceptible to acetaminophen induced liver injury, but mdivi-1 induced these animals to be much more sensitive to acetaminophen [121].

In addition, the nervous system is highly sensitive and significantly alters its mitochondrial function after undergoing acute injury or long-term stimuli. Recent studies state that mutations of Drp1 in human result in disturbance of mitochondrial fission and lead to various diseases. Hyper-phosphorylation of Drp1 at Ser-637 induced abnormal mitochondrial dynamics in somatic cells isolated from hereditary spastic paraplegia patients; this disturbance is mediated by impaired interactions between receptor expression enhancing protein 1(REEP1) and PGAM5 [53]. In autosomal recessive spastic ataxia of Charlevoix Saguenay patients, neurons demonstrate reduced ability to recruit Drp1 and poor mitochondrial health [60]. Drp1 mutation also results in postnatal microcephaly, developmental delay, and pain insensitivity via impairment of mitochondrial fission and mitochondrial respiratory function [63]. In addition, mitochondrial functions are changed in the brains of individuals with specific neurodegenerative disorders. There is still controversy over Drp1-mediated mitochondrial fission in Alzheimer’s disease. Drp1 is termed a crucial factor for mitochondrial dynamics in patients with Alzheimer’s disease [122], and their brain tissues demonstrated elongated interconnected mitochondria; however, this is not associated with altered translocation of Drp1 but with reduced GTPase activity [123]. P110 reduced mitochondrial fragmentation and corrected mitochondrial dysfunction fibroblasts from patients with Hodgkin’s disease and Parkinson’s disease [59,124]. Camptothecins, which specifically target DNA topoisomerase I and serve as anticancer drugs in clinical trials [66], are also demonstrated to decrease the levels of Drp1 and parkin in neurons; overexpression of Drp1 or parkin in neurons improves neuronal viability and maintains mitochondrial morphology, thus promoting resistance to apoptotic stimuli [125]. Furthermore, loss of parkin alone is not sufficient to decrease the mitochondrial connectivity but parkin is effective in the absence of Drp1 [126]. PP2A, a neuron-specific regulatory subunit, activates Drp1 by dephosphorylating Ser-656, which is targeted by the neuroprotective protein kinase, whereas blockage of Drp1 dephosphorylation and mitochondrial fragmentation increase the apoptosis rates of cultured hippocampal neurons [127]. Nanoceria, which acts as an antioxidant by reversibly binding oxygen and shifting between the Ce^3+^ and Ce^4+^ forms [128], can not only reduce peroxynitrite induced ROS and protein tyrosine nitration in neurons, but also reduces endogenous peroxynitrite and neuronal cell death rates via inhibition of Drp1 Ser-616 hyperphosphorylation [63]. Most studies have sought to prove that the pathogenesis of nervous system degenerative diseases is closely linked to Drp1-dependent mitochondrial fission, as has been observed in previous acute injury models. Since aging cells are observed in various neurodegenerative diseases, how to inhibit the pathological processes is quite important for treating these diseases. Leucine-rich repeat kinase 2 and Drp1 partially co-localize to induce defects in the mitochondrial dynamics of neurons and consequently lead to Parkinson’s disease [129]. In Alzheimer’s disease, GSK3β increases Drp1-dependent GTPase activity and results in modified neurons being more vulnerable to apoptotic signals; in contrast, blocking GSK3β-induced Drp1 phosphorylation efficiently protects neurons against apoptosis [130]. Partially reduced Drp1 significantly decreases Aβ production and effectively maintains mitochondrial dynamics in Alzheimer’s disease neurons and APP transgenic mice (Tg2576 line) [45]. Lentiviral transduction with wild-type STAT2 rescued the deficiency in STAT2-deficient patient-derived fibroblasts via upregulation of phosphorylation of Drp1 at Ser-616 and maintenance of mitochondrial length [53]. Additionally, Drp1 also plays critical roles in other relatively slowly progressing diseases. For example, airway smooth muscle cells from asthmatic patients exhibited substantial morphological defects and showed increased Drp1 expression [131]. Drp1 protein levels are profoundly diminished in the peripheral blood lymphocytes of systemic lupus erythematosus patients [57]. In diabetes models, inhibition of Drp1 by Drp1-K38A attenuates fatty acid-induced mitochondrial fragmentation in stressed β-cells [132]. Rhein preserves mitochondrial ultrastructure by ablating cellular ROS and decreasing Drp1 activity in hyperglycemia [133]. In addition to these small molecules, aerobic exercise training significantly downregulated the phosphorylation level of Drp1 at Ser-616 and increased fat oxidation and insulin sensitivity in insulin-resistant human skeletal muscle cells [56].

Intriguingly, mitochondrial fission precedes other hallmarks in multiple tumors [134]. Because tumor cells are difficult to selectively clear, and there is high mortality among end-stage cancer patients, there is a great need to find effective and safe methods to save the lives of patients. After Drp1 activity was inhibited in brain-tumor-initiating cells, the tumorigenicity, both in vitro and in vivo, was attenuated through activation of AMPK [135]. Blockage of Drp1 activity also kills thyroid cancer cells and brain-tumor-initiating cells by altering the migration or invasion ability of tumor cells [135,136]. Loss of Drp1 suppresses growth of hepatocellular carcinoma cells through suppression of the p53/p21 and NF-κB/cyclin pathways [137]. In recent years, various protocols for inhibition of Drp1 activity have been applied to the experimental and clinical tests in cancer animal models or patients. Mdivi-1 significantly increases mitochondrial ROS production, mitochondrial mass, and cardiolipin oxidation and it selectively sensitizes cancer cells to apoptosis, providing favorable curative effects [138]. Since cofilin S3E and Drp1 S637D mutants significantly suppressed mitochondrial fission and mitochondria-dependent apoptosis, erucin inhibits tumor growth through mitochondrial translocation of cofilin and Drp1 in a breast cancer cell xenograft mouse model [139]. In addition, Drp1 RNA interference increases the apoptotic rates of human lung and colon cancer cells through inhibition of Drp1-dependent mitochondrial fission [140]. Sodium butyrate significantly reduces the cyclin B1–CDK1 complex and phosphorylates Drp1 and consequently induces apoptosis of human colorectal cancer cells [141]. After knockdown of tumor necrosis factor receptor-associated protein 1, the expression levels of Drp1 and Mff are downregulated, whereas the expression of fusion proteins is not changed in neuroblastoma cells and glioma cells [142]. In addition to these gene modifications in Drp1-related pathways, co-culture with mesenchymal stem cells induces T-cell acute lymphoblastic leukemia cells to keep well-maintained mitochondrial dynamics, mitochondrial ROS levels, and chemoresistance via extracellular signal-regulated kinase activation-mediated phosphorylation of Drp1 at residue Ser-616 [66]. Although altered expression of Drp1 efficiently increases apoptosis in various malignant cells, Drp1-dependent mitochondrial fission alone is not sufficient to cure diffuse large B-cell lymphoma [143]. Currently, the role of Drp1 in malignant tumors is uncertain, and the detailed regulatory mechanisms remain to be determined. Furthermore, new drugs targeting the mitochondrial fission pathway should be developed.

## 7. Conclusions

As highly dynamic organelles, mitochondria play crucial roles in cell survival as well as cell death by altering their morphology, mass, and function in response to stress and physiological conditions. Although mitochondria are the smallest semi-autonomous system in mammals, mitochondrial morphology, distribution, and function are maintained by the balanced regulation of mitochondrial fission and fusion. Drp1 and its specific receptors (Drp1, Mff, MiD49/51, and human Fis1) cooperatively regulate the fission process. Assembly, hydrolysis, disassembly, and post-translational modifications of Drp1 are also indispensable for the regulation of Drp1 activities in biological dynamics. In addition, because post-translational modifications at different sites may exert effects on cells or tissues considerably differently, Drp1 should be modified at specific sites in order to regulate Drp1 activity. Although mitochondrial fusion is infrequently investigated in recent studies, it has been demonstrated to cooperate with mitochondrial fission machinery in maintaining the health of cells or diminishing apoptosis rates in vivo and in vitro. Interestingly, regulation of this critical protein also takes part in the apoptosis progress of various tumor cells and may greatly improve the survival rates of end-stage cancer patients. Since the composition of the fission apparatus may vary according to disease states, different species, and even different individuals, future studies are necessary to elucidate the complex interplay and coordination between Drp1 and various diseases in human. Developing a better understanding of the mitochondrial fission and fusion machinery should provide an avenue to better address the numerous pathological processes to which mitochondrial homeostasis contributes. Advantage remodeling will be controlled through quantitative balance between mitochondrial fission and fusion processes. According to current studies, several pharmacological agents effectively reverse the impairment of mitochondrial dynamics, but the related pharmacokinetics and toxicology profiles need to be further investigated for clinical usage.

## Figures and Tables

**Table 1:** Phosphorylated dynamin-related protein 1 (Drp1) at different sites and the correlated regulative ways for mitochondrial fission in mammals in vivo and in vitro.

Cell Type	Stimuli	Sites	Regulation	Effects	References
Cardiomyocytes	Anoxia-reoxygenation injury	Ser-616	↑	Up-regulation of ROS production and mitochondrial fission	[13]
Cardiomyocytes	Anoxia-reoxygenation injury	Ser-637	↓	Up-regulation of ROS production and mitochondrial fission	[13]
Vascular smooth muscle cell	Glucagon-like peptide-1	Ser-637	↑	Stimulation of mitochondrial fusion and inhibition of vascular smooth muscle cell dedifferentiation	[39]
Hippocampal neurons	Ca^2+^-dependent protein kinase Iα	Ser-600	↑	Mitochondrial fragmentation	[39]
Mouse podocytes and endothelial cells	Hyperglycemia	Ser-600	↑	Recruitment of Drp1	[52]
Human podocytes and endothelial cells	Hyperglycemia	Ser-637	↑	Recruitment of Drp1	[52]
STAT2-deficient patient derived fibroblasts	Lentiviral transduction with wild-type STAT2	Ser-616	↑	Maintenance of mitochondrial length	[53]
Pulmonary vessels	Cdk1/cyclin B	Ser-616	↑	Pulmonary arterial remodeling	[54]
Smooth muscle cells	Angiotensin II or hydrogen peroxide	Ser-616	↑	Proliferation and migration	[55]
Human skeletal muscle cells	Aerobic exercise	Ser-616	↓	Up-regulation of fat oxidation and insulin sensitivity	[56]
Cardiomyocytes	Pim-1	Ser-637	↑	Maintenance of a reticular mitochondrial phenotype under ischemia condition	[57]
Cardiac myocytes	Dominant-negative forkhead box O3a	Ser-637	↑	Up-regulation of maladaptive cardiac atrophy genes	[58]
Cardiac myocytes	FK506 treatment prior to IR	Ser-637	↓	Preservation of cardiac function	[59]
Post-mitotic neurons	Cyclin-dependent kinase 5	Ser-616	↓	Modulation of mitochondrial morphology	[60]
Neural cells	Mild hypothermia	Ser-616	↓	Preservation of neural cells integrity	[61]
Neural cells	PTEN-induced putative kinase 1	Ser-616	↓	Neuronal survival	[62]
Neuronal cell	Nanoceria	Ser-616	↓	Reduction of ROS, protein tyrosine nitration, endogenous peroxynitrite and cell death rates	[63]
Hippocampal cells	Wnt-5a	Ser-616	↑	Up-regulation of intracellular and mitochondrial calcium	[64]
Hippocampal cells	Wnt-5a	Ser-637	↓	Up-regulation of intracellular and mitochondrial calcium	[64]
HeLa cells	Depletion of death associated protein 3	Ser-637	↓	Increased apoptotic sensitivity	[65]
T-cell acute lymphoblastic leukemia cells	Mesenchymal stem cell co-culture	Ser-616	↑	Maintenance of mitochondrial dynamics, mitochondrial ROS levels, metabolic switching and chemoresistance	[66]

↓, Down-regulation; ↑, Up-regulation; FK506, tacrolimus; PTEN, phosphatase and tensin homolog; ROS, reactive oxygen species; IR, ischemia–reperfusion.

**Table 2:** Drp1-dependent mitochondrial fission and development in vivo and in vitro.

Basal Background	Treatments for Drp1	Effects	Targets	Species	References
In vivo	Loss	Impairment of Ca^2+^ signaling and intercellular communication	Aged oocytes	Mouse	[76]
	Unaltered	Significant increase in the Mfn2-to-Drp1 ratio; longer and more branched intermyofibrillar mitochondria	Aged muscles	Mouse	[77]
	Loss	Death	Mouse at day 12.5 during embryonic period	Mouse	[78]
	Loss	Left ventricular dysfunction and lethal heart defects	Cardiomyocytes	Mouse	[79]
	Inhibition	Inhibit the p53 mediated apoptotic pathways	Neurons in MPTP animal model	Mouse	[80]
	Cardiac-specific loss	Impair left ventricular function and lead to death within 13 weeks	Cardiomyocytes	Mouse	[79]
	Loss	Impair neural tube formation and lead to death at embryonic day 11.5	Neural cells	Mouse	[81]
	Overexpression	Impair postnatal muscle growth and reduce mtDNA quantity and the growth hormone pathway	Muscle	Transgenic mouse line	[82]
In vitro	Loss	Negatively influence terminal differentiation, particularly in the neurogenetic differentiation	ESCs	Mouse	[83]
	Loss	Augmentation of the cyclin E pool for attenuating cell proliferative rates	Embryonic fibroblasts at low density	Mouse	[84]
	Loss	Aberrant cell proliferation	Embryonic fibroblasts at high density	Mouse	[84]
	Loss	Impair myogenic differentiation potency	Myogenic precursor cells	Mouse	[85]
	Loss	Decrease in aerobic metabolism, calcium flux and proliferation	Ductal smooth muscle cells	Rabbit	[86]
	Loss	Increase mitochondrial length and lead to cell death	Cortical neurons	Mouse	[87]

MPTP, 1-methyl-4-phenyl-1,2,3,6-tetrahydropyridine; ESCS, embryonic stem cells.

## Abbreviations

ATP	Adenosine triphosphate
Drp1	Dynamin-related protein 1
GED	C-terminal GTPase effector
MOM	Mitochondrial outer membrane
ER	Endoplasmic reticulum
ROS	Reactive oxygen species
Mff	Mitochondrial fission factor
MiD51	Mitochondrial elongation factor 1
MiD49	Mitochondrial elongation factor 2
ADP	Adenosine diphosphate
Fis1	Mitochondrial fission 1
Mfn	Mitofusion
MIM	Mitochondrial inner membrane
Opa1	Optic atrophy 1
AMPK	Adenosine monophosphate-activated protein kinase
PKA	Protein kinase A
AKAP1	A kinase anchoring protein 1
CDK	Cyclin-dependent kinase
PP2A	Protein phosphatase 2A
GSK	Glycogen synthase kinase
FK506	Tacrolimus
PTEN	Phosphatase and tensin homolog
SENP	Sentrin/SUMO-specific protease
KO	Knockout
MPTP	1-Methyl-4-phenyl-1,2,3,6-tetrahydropyridine
mtDNA	Mitochondrial DNA
Gfer	Growth factor erv1-like
ESCs	Embryonic stem cells
MEFs	Mouse embryonic fibroblasts
OCRs	Oxygen consumption rates
PGAM5	Phosphoglycerate mutase 5
H_2_O_2_	Hydrogen peroxide
IR	Ischemia–reperfusion
PINK1	PTEN-induced novel kinase 1
RGC	Retinal ganglion cell
REEP1	Receptor expression enhancing protein 1
